# On the Quantification of SSVEP Frequency Responses in Human EEG in Realistic BCI Conditions

**DOI:** 10.1371/journal.pone.0077536

**Published:** 2013-10-18

**Authors:** Rafał Kuś, Anna Duszyk, Piotr Milanowski, Maciej Łabęcki, Maria Bierzyńska, Zofia Radzikowska, Magdalena Michalska, Jarosław Żygierewicz, Piotr Suffczyński, Piotr Jerzy Durka

**Affiliations:** 1 Faculty of Physics, University of Warsaw, Warsaw, Poland; 2 Department of Psychology, University of Social Sciences and Humanities, Warsaw, Poland; 3 Department of Molecular and Cellular Neurobiology, Nencki Institute of Experimental Biology, Warsaw, Poland; University College of London – Institute of Neurology, United Kingdom

## Abstract

This article concerns one of the most important problems of brain-computer interfaces (BCI) based on Steady State Visual Evoked Potentials (SSVEP), that is the selection of the a-priori most suitable frequencies for stimulation. Previous works related to this problem were done either with measuring systems that have little in common with actual BCI systems (e.g., single flashing LED) or were presented on a small number of subjects, or the tested frequency range did not cover a broad spectrum. Their results indicate a strong SSVEP response around 10 Hz, in the range 13–25 Hz, and at high frequencies in the band of 40–60 Hz. In the case of BCI interfaces, stimulation with frequencies from various ranges are used. The frequencies are often adapted for each user separately. The selection of these frequencies, however, was not yet justified in quantitative group-level study with proper statistical account for inter-subject variability. The aim of this study is to determine the SSVEP response curve, that is, the magnitude of the evoked signal as a function of frequency. The SSVEP response was induced in conditions as close as possible to the actual BCI system, using a wide range of frequencies (5–30 Hz, in step of 1 Hz). The data were obtained for 10 subjects. SSVEP curves for individual subjects and the population curve was determined. Statistical analysis were conducted both on the level of individual subjects and for the group. The main result of the study is the identification of the optimal range of frequencies, which is 12–18 Hz, for the registration of SSVEP phenomena. The applied criterion of optimality was: to find the largest contiguous range of frequencies yielding the strong and constant-level SSVEP response.

## Introduction

Brain responses to repetitive sensory stimulus have been studied for decades. For instance Regan [1] has observed that a rapidly repeating stimulus, such as a flickering light of certain frequency, may induce response in corresponding frequencies (that of stimulation and higher harmonics) in the EEG recorded over visual areas of the scalp. These brain responses have been named steady-state visual evoked potentials (SSVEP). This phenomenon is commonly used in Brain-Computer Interface (BCI) systems [2]. A graphical interface of the SSVEP-based BCI system usually consists of different commands, e.g. letters or symbols, that flicker at specific frequencies. User pays attention to a particular flickering command, while ignoring others, which induces SSVEP with the corresponding frequency. BCI system identifies the user intention by quantifying and classifying SSVEP. Proper choice of flicker frequencies and accurate estimation of the response magnitude are critical for BCI. Although it is generally acknowledged that the SSVEP response depends on the frequency of the stimulation, there are relatively few studies investigating this relation in detail. Regan [3] has shown that the dependence of the amplitude of SSVEP on the flicker frequency generally exhibits three distinct maxima. The ‘low-frequency’ response with a maximum around 10 Hz, the ‘medium-frequency’ response in 13–25 Hz range and ‘high-frequency’ response in 40–60 Hz range. Similar results have been subsequently obtained in other studies.

In [4], a representative dependence of SSVEP amplitude on frequency response of one subject exhibits three maxima centered on 15, 31 and 41 Hz. Pastor [5] investigated the EEG oscillatory responses to flicker stimulation for selected frequencies in the 5–60 Hz range. The response amplitude was largest at 15 Hz in the occipital area and at 25 Hz in the frontal areas. Herrmann [6] investigated the EEG responses to flicker stimulation in the frequency range 1–100 Hz with 1 Hz resolution. His main finding was that the brain exhibits resonant frequencies around 10, 20, 40 and 80 Hz. The relative magnitudes of response frequencies were not the main objective of the study, hence the results do not pertain directly to BCI systems, e.g., the curve representing average response magnitude across 10 subjects was not smooth and exhibited several maxima and minima. The standard error of the mean was not provided hence it is not possible to assess statistical significance of the peaks.

Although the dependence of response magnitude on stimulation frequency has been investigated in several studies, and is commonly used to guide the selection of stimulation frequencies, there is no consensus regarding the optimal frequencies for the SSVEP-based BCIs. This is reflected in very diversified frequencies adopted in various studies. Some authors used narrow ‘low-frequency’ band, e.g. 6.666–8.571 Hz [7], 5–9.9 Hz [8], while others used medium frequency range, e.g. 14–18 Hz [9] or even higher frequencies e.g., 27–43 Hz [10]. The application of frequencies from the alpha band (8–13 Hz) is also not consistent across studies. While in some BCI applications, frequencies from the alpha band are excluded (e.g., 6, 7, 8 and 13 Hz [11]), they are included in some other studies (e.g. 6.67, 7.50, 8.57, 10.00, 12.00 Hz [12]). The widespread diversity of the stimulation frequencies across studies indicates that there is still a need to find out which frequencies may provide an optimal performance of the SSVEP-based BCI.

The goal of this study is to provide a link between fundamental research and its applications in designing knowledge-based BCI systems with maximum performance. For this purpose we investigate the SSVEP responses in the wide range of responsive stimulation frequencies, using a 4-class SSVEP-based BCI. To the best of our knowledge, this topic has not been investigated in detail previously, in the settings corresponding to real BCI application.

## Materials and Methods

### Subjects and Data Collection

Ten subjects participated in this study. All right–handed, 5 males and 5 females, mean age: 28.7 years, range: 24–41. EEG was recorded by means of 19 Ag/Ag-Cl electrodes placed on the surface of the scalp according to the international 10–20 system. All the electrodes from this system, except Fp1 and Fp2, were used to further analysis. The ground electrode was placed over the clavicle of the subject and the reference electrodes were placed over the left and right mastoid (M1 and M2). The impedance of the electrodes was below 5 k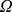. The signal was acquired by Porti7 (TMSI) amplifier with sampling frequency 1024 Hz.

### Visual stimulation

Subjects were seated on a comfortable chair, in a dim room. Visual stimulation was delivered using a custom made BCI stimulator [13], placed at 100 cm in front of the subject. It consisted of liquid crystal display (LCD) backlit by an array of LEDs. The LCD (19.5 by 35 cm) was divided into four square fields, 4.5 cm by 4.5 cm each in 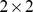 arrangement, separated by 4 cm of blank space. The size of the single square field, observed from the distance of 100 cm, was 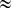
2.6 degrees of visual angle and the distance between the centers of two adjacent fields was 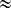
4.9 degrees. This geometry implies that that if the subject focused on the center of the target field the target stimulus would be situated in foveal vision and the neighbouring ones would be localized in peripheral vision. Such localization of flickering fields should reduce the interference of neighbouring stimuli in the EEG signal [14]–[16] and the distraction of attention of the subject, yet it requires only relatively small eye movement to change the attended field. The luminance of stimuli emitted by single square field was 30 lx. Each LED highlighted a single field of the display. The LEDs were controlled by a micro-controller (MCU). The MCU drove the LEDs with square wave of stable frequency and duty cycle equal to 0.5. Each LED could flicker with a different frequency.

### Experimental Paradigm

**Figure 1:**
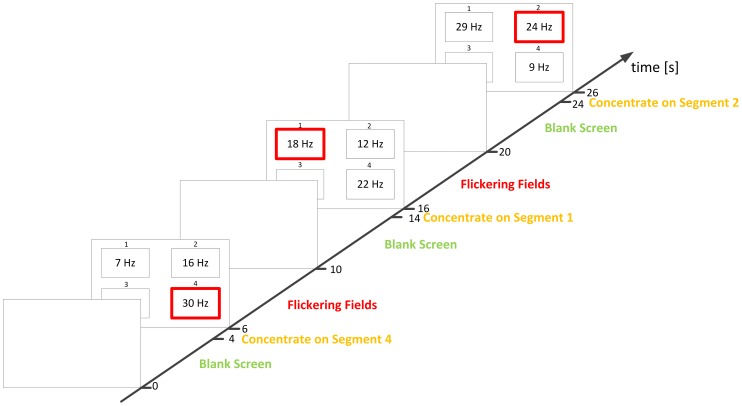
Scheme of the experimental paradigm. In resting epochs the screen remains blank, whereas during stimulation each field flashes with the indicated frequency. The task of the subject is to focus attention on the field indicated by a voice command issued two seconds before the stimulation onset. Indicated field is marked red.

**Figure 2:**
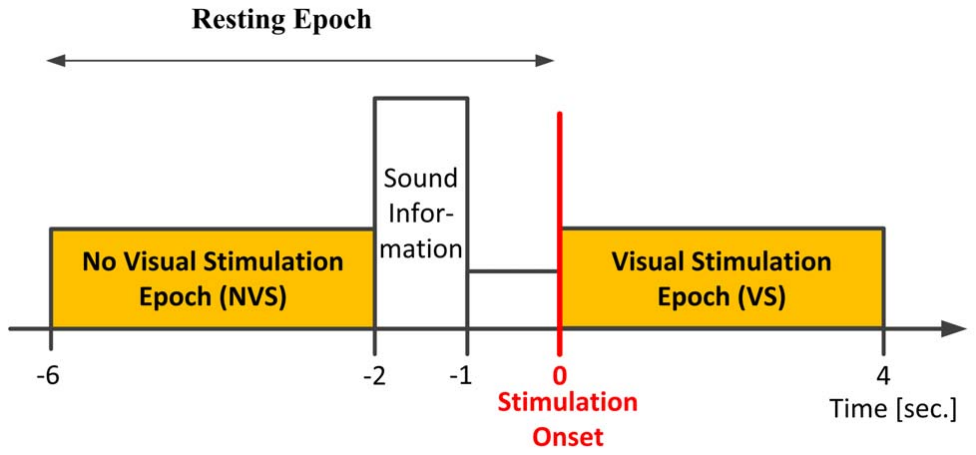
Scheme of a single experimental trial. Resting period lasted 6(at 0 s.), the subject was instructed by a voice command to which of the fields he or she should attend. The signals from −6 to −2 s. of the resting epoch and signal from 0 to 4 s. of the stimulation epoch were selected for further analysis as No Visual Stimulation Epochs and Visual Stimulation Epochs, respectively.

The schematic sequence of events is presented in Fig. 1. The experiment consisted of 4 s long stimulation periods interleaved by 6 s long resting periods. During the resting period the LCD panel was turned off. During the stimulation intervals, in order to create experimental conditions corresponding to SSVEP paradigm used in BCI systems, all four fields were simultaneously active, each flickering with different frequency. Two seconds before stimulation onset the subject was instructed by a voice command to which of the four fields he or she should attend. Thus we consider two experimental conditions: Visual Stimulation (VS) with given frequency 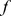 and No Visual Stimulation (NVS) (Fig. 2). Further data analysis will consider mainly the VS condition. Both the position of the attended segment and its flickering frequency were randomly selected to avoid habituation. The subject was stimulated 50 times with each integer frequency between 5 and 30 Hz, i.e. 5, 6, 7,. 29, 30 Hz. Due to the length of the test, the experiment was divided into five sessions of 40 minutes. In each session the subject was stimulated with 10 repetitions of each frequency. For each subject, the full test was split into two meetings (two and three sessions, or vice versa). The length of the break between sessions during one day usually lasted about 15 minutes.

### Ethics statement

The study was approved by the Research Ethics Committee at University of Social Sciences and Humanities in Warsaw, Poland. All subjects declared the absence of neurological or mental illnesses, and were screened against the photosensitive epilepsy by the standard clinical EEG test. Informed, written consent was obtained from all of the subjects.

### Data analysis

There are two important issues concerning the assessment of the magnitude of the SSVEP response. The first comes from the fact that the EEG signal contains both the activity related to visual stimuli and activity related to other processes. Second issue relates to the definition of the measure, which would quantify the SSVEP in an efficient way, relevant to the prospective application. Because of these issues, application of appropriate signal preprocessing and processing algorithms to extract the relevant activity is the central point of any SSVEP-based BCI. In the following sections we focus on techniques which are feasible in potential BCI application, that is those, that after a calibration session can be applied on-line.

#### Extraction of resting and stimulation epochs

From the EEG signal we extract segments of interest: 4 s long epochs of signal preceding the stimulation onset by 2 s, denoted as 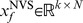, and 4 s long epochs of signal during the stimulation with frequency 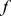, denoted as 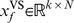, where 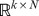 denotes a 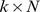 real number matrix, 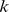 is the number of channels and 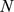 is the number of samples (Fig. 2).

#### Spatial Filtering

The recorded signal contains response to light stimulation and background EEG activity. To increase the ratio of the SSVEP magnitude to the magnitude of the background activity, we applied Common Spatial Pattern filter (CSP) [17]–[20]. This filter, in case of two multichannel time-series, yields a linear combination of the original channels such that the variance of one of the resulting signals is maximized for one of the time-series and simultaneously it is minimized for the other time-series. It is expected, that the most prominent changes in EEG signal, during the flickering stimulation, will be observed in the frequency of stimulation. All data segments 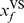 were band-pass filtered by means of 1-st order Chebyshev Type II filter with pass-band centered at frequencies of interest 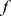. The width of the pass-band was 2 Hz (from 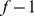 Hz to 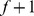 Hz). The time series filtered around frequency 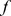 are denoted as 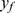.

The CSP transformation matrix 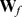 was estimated separately for each stimulation frequency 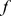, since their spatial filters might not be identical. The matrix 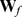 can be obtained as a solution of the generalized eigenvalue problem [21]:
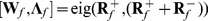
(1)where: 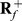 is the mean covariance matrix estimated by ensemble averaging of the single trial covariances matrices of 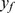 – it describes the covariance structure of the signal of interest, and 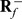 is the mean covariance matrix estimated by ensemble averaging of the single trial covariances matrices of 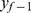 and 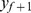 – it describes the covariance structure of the background activity in adjacent frequencies; 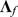 is a diagonal matrix of eigenvalues and 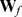 is a full matrix whose columns are corresponding eigenvectors so that 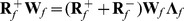. Next, the original signals 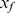 were transformed by the estimated CSP filter:



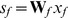
(2)The CSP filter, like other Blind Source Separation techniques, does not guarantee that the transformed signals 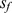 contain the brain electrical activity separated into physiologically meaningful components. But, by the construction of the CSP filter, the channel corresponding to the highest eigenvalue in 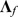, has the maximal variance for the 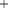 condition and lowest variance for the 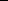 condition. Further, the covariance matrices were estimated by ensemble averaging, thus the considerable contribution to 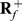 and 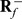 will be derived from the EEG activity which has persistent covariance structure over the trials. That's why we assume that channel corresponding to the highest eigenvalue in 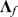, denoted as 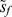, will contain the activity related to SSVEP. The validation of this assumption will be presented in the Results section.

#### Spatial Patterns

It is very important to understand the relationship between the spatial filters, represented by each row of the matrix 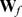 and the corresponding spatial patterns. The CSP filter, in given output channel, produces a signal containing the activity uncorrelated to activity in other output channels. Assuming that this activity is generated by some sources, one can compute the projection of their activity on the scalp. From (2) one gets:
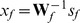
(3)


Each column of matrix 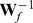 gives the contribution of the corresponding source to the EEG signal and it is called spatial pattern of signal 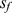. It can be visualized as a plot showing its spatial distribution. The comparison of the obtained spatial filter and spatial patterns will be shown in the Results section.

#### Spectral power

The signals 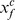, (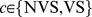) corresponding to given stimulation frequency 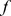 (both NVS and VS epochs) were first transformed by relevant CSP filter, then the spectral power estimates, 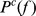, were computed for the channel 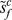, which corresponds to the highest eigenvalue in 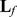. 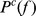 is obtained by evaluating the spectral power at frequency 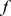 by means of periodogram with Tukey window with parameter equal to 0.1. For the convenience of between subject comparison the average spectra were normalized to the maximal value obtained for the VS condition for a given subject:
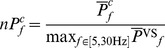
(4)where: 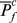 is the 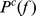 averaged over experiment realizations with given stimulation frequency 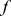 for a given subject.

#### Signal to Noise Ratio

Spectral power of EEG decreases with frequency. This property implies that response to high-frequency stimulation has less absolute power than response to low-frequency one. Therefore SSVEP can be better quantified as a relative increase of power at the stimulation frequency, with respect to its baseline value. The quantity, often used to measures the relative increase or decrease of the EEG power, is Event Related Spectral Perturbation [22]. However in context of on-line computations, relying on baseline values is not convenient. A more practical approach, especially applicable for SSVEP, was proposed in [23]. It measures the activity level at a given stimulation frequency (regarded as signal level) with respect to the level of activity in adjacent frequencies (regarded as noise level). Expressed as a ratio of corresponding powers this measure is an estimator of Signal-to-Noise-Ratio (SNR) for a given realization of experiment:
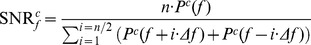
(5)


we used: 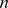 = 6, 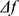 = 0.25 Hz. This estimator of SNR was used as a feature for classification in BCI e.g. in [28]. In order to compare the average SNR between subjects we propose to normalize it for each subject by its maximal value:
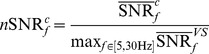
(6)where: 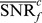 is the 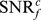 averaged over experiment realizations with given stimulation frequency 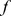 for a given subject.

## Results

### Spatial Filters and Spatial Patterns

**Figure 3:**
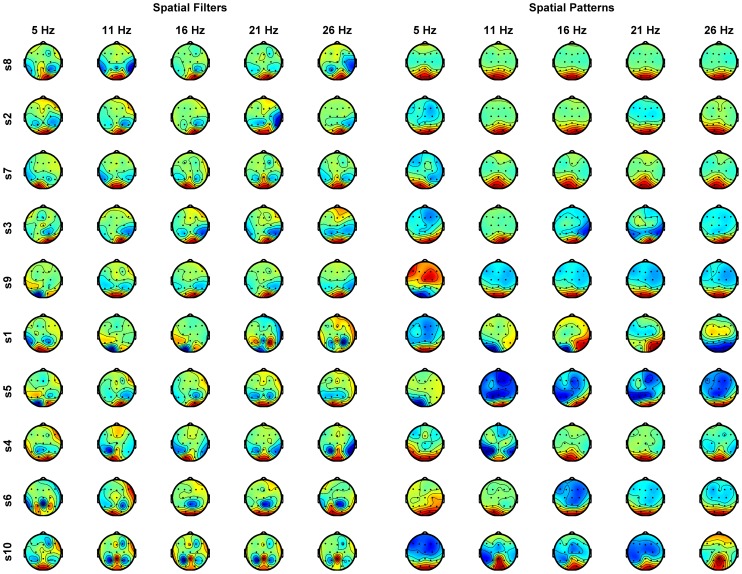
Visualisation of the spatial filters and spatial patterns corresponding to the highest eigenvalue (eq. 2 and 3). Four left columns – spatial filters, four right columns – spatial patterns at stimulation frequencies: 5 Hz, 11 Hz, 16 Hz, 21 Hz and 26 Hz. Each row presents results for one subject. Color code: red positive, blue negative values.

Examples of spatial filters and spatial patterns corresponding to the highest eigenvalue for each subject and for subset of stimulation frequencies is presented on the Fig. 3. One can observe, that the spatial patterns in most cases have clear dipolar form with extremum at the parietal and occipital electrodes, compatible with a hypothetical SSVEP source located in visual cortex. In most cases the pattern is symmetric, with extremum around electrodes O1 and O2 (e.g. subject S8), but in some cases it is asymmetric, with extremum either around the electrode O2 (e.g. subject S1 at frequency 21 Hz) or electrode O1 (e.g. subject S1 at frequency 11 Hz). Only five subjects (S8, S2, S7, S3, S9) have almost constant spatial pattern across frequencies. For the rest of the subjects the spatial pattern varies with frequency.

The spatial patterns, although not constant, are generally consistent (up to sign) within and between subjects. But the spatial filters show much larger variability. This diversity results from different activity not related to SSVEP in different frequency bands (e.g. background EEG, muscle activity). The aim of the spatial filters is minimization of this additional activity in favor of SSVEP, thus the obtained spatial filters differ for the different stimulation frequencies.

### SSVEP Frequency Response

#### Course of the response curve

**Figure 4:**
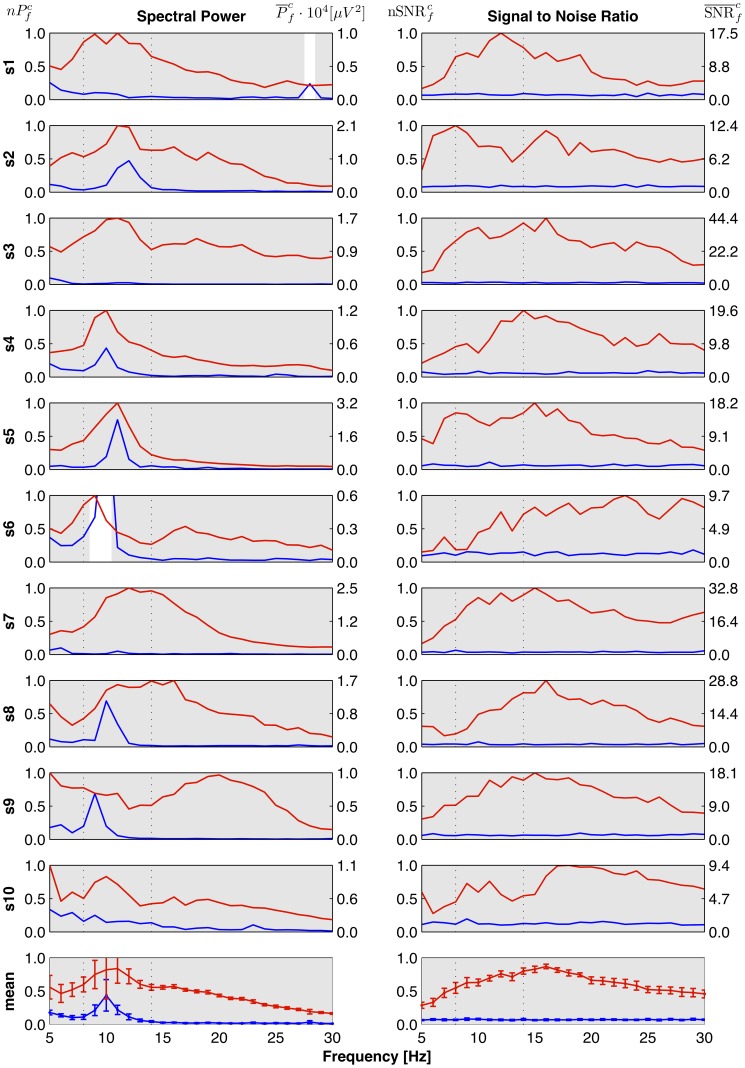
SSVEP frequency response curves. Columns correspond to the method of SSVEP evaluation: Left – spectral power, right – Signal to Noise Ratio. Rows 1–10 correspond to individual subjects, last row shows the response averaged across 10 subjects. Horizontal axis – stimulation frequency. Vertical axes have two scales. In the left column: left scale – normalized power 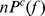, right scale – absolute mean power 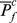. In the right column: left scale – values of 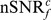, right scale – values of 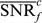. Error bars indicate the RMS error. Each plot presents two curves: the blue line shows values obtained for NVS, and the red one for VS condition. The gray regions in rows 1–10 mark the frequencies, where the given measure (column) gives significantly higher result for VS than NVS epochs for the a given subject (row) – results of within-subject level tests. The gray color on the plots in the last row means significant reactive frequencies for the population. Dotted vertical lines at 8 Hz and 13 Hz mark the range of the 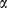-band. Note: For S6 in left column the blue line exceeds the shown range to reach the value of 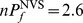.

SSVEP frequency responses obtained by means of normalized spectral power 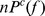 are shown in the left column of Fig. 4 for all individual subjects (rows 1–10); the last row shows the responses averaged across subjects. The maxima of the response curve correspond to the presence of three frequency ranges of EEG rhythms: 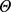 (5–7.5 Hz), 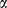 (8–13 Hz) and 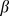 (above 14 Hz) [24]. For half of the subjects (S1, S2, S3, S4, S5 and S10) there is a pronounced peak in 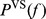 centered in the 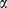 band. Only for some of them it corresponds exactly to the peak in 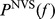 (S2, S4 and S5). Two of the subjects have a broad peak in 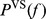 extending from 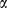 to 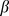 band (S7, S8), and two of them have the peak shifted towards 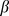 range (S6, S9). In the average response curve, one can see that the highest values of 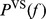 are obtained for the 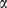 range, but they have considerably higher root-mean-square (RMS) error than these in the 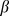 range.

The nSNR measure gives ratios of power at the stimulation frequency to the mean power at adjacent frequencies. This makes it less sensitive to the fact that with the increase of frequency the measured bioelectrical brain activity is more attenuated. As a result for most of the subjects we observe slower decrease of the SSVEP response with frequency, and in general the response curve is much more leveled (cf. Fig. 4 right vs left panel in each row). The response curve averaged over subjects (Fig. 4 bottom panel) reveals that there is a broad peak ranging from 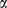 to 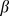 band, with its peak located around 16 Hz. With respect to the average response measured by normalized spectral power the peak is shifted from 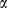 to 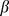 band.

#### Reactive frequencies

We performed two levels of statistical tests. The first level, within subjects, seeks an answer: at which of the stimulation frequencies the response evaluated by given measure (either 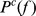 or 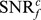) is higher for the VS than for the NVS epochs. For this test we applied one sided Wilcoxon test with False Discovery Rate (FDR) [25] correction for multiple comparison; the maximum FDR level (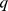-value) was set to 5%. From this level of analysis for each subject we obtain list of frequencies for which the null hypothesis was rejected. This are subject's reactive frequencies.

The second level, between subjects, seeks an answer whether a given stimulation frequency is reactive in the population, i.e. can we reject the hypothesis that for the considered frequency the given measure yields higher result for the VS than for the NVS epochs in not more than half of the population. We treat the first level test results for given frequency across subjects as a Bernoulli process (with sequence of length equal to the number of subjects and number of successes equal to the number of subjects for whom the considered frequency is the reactive one). We compute the probability of obtaining such or more extreme sequence from Bernoulli distribution with parameter equal to 0.5 and correct it for multiple comparison with FDR (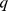-level
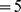
%).

In Fig. 4 in rows 1–10, the significant – according to the within-subject level tests – SSVEP responses are marked in gray. The results show that normalized spectral power measure does not detect the significant SSVEP response only for subject S6 for stimulation frequencies within 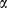 band, and for subject S1 in the 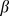 band. The last row shows the mean responses averaged across subjects. In this panel, the gray bars indicate significant results of the second level statistical test. We see that for all tested frequencies the hypothesis that SSVEP reaction will be significant in not more than half of the population can be rejected. In this sense, we can state that all frequencies are reactive according to both spectral power and SNR measure.

### Optimal frequencies

**Figure 5:**
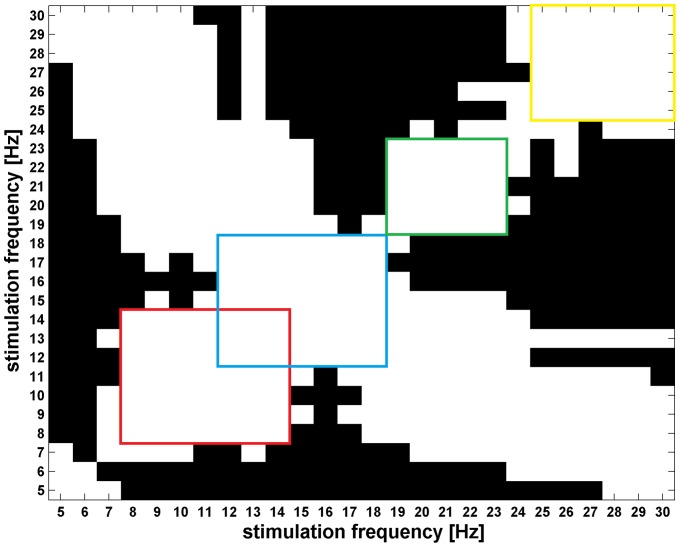
Test of magnitude of nSNR response for pairs of frequencies. White pixel indicate a pair of stimulation frequencies (one from horizontal, and one from vertical axis) which do not show significant differences in mean nSNR response, black pixel indicates that the difference is significant. Color squares indicate contiguous frequency ranges which pairwise have equal mean responses.

**Figure 6:**
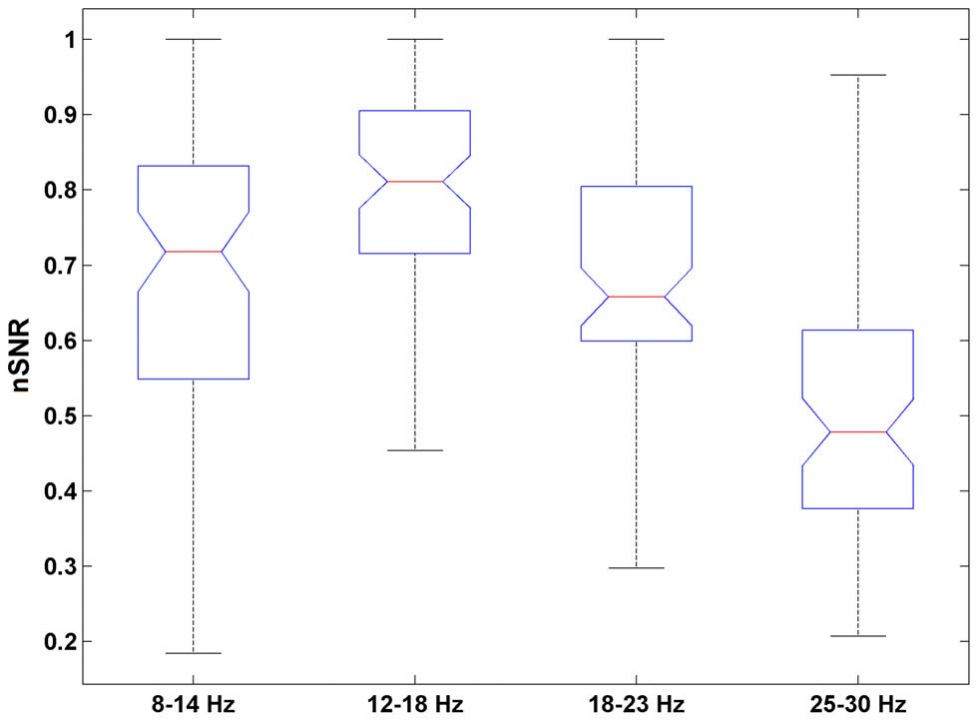
Distribution of nSNR responses in selected frequency ranges. Each box-plot shows median (middle red line) together with its 95% confidence interval (notches). The lower and upper edge indicate 25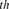 and 75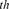 quantile, the whiskers show the span the data.

The next, more detailed question is which frequency range, on the population level, is optimal for SSVEP detection. The optimality criteria applied is that the response is the largest and similar for different frequencies within a contiguous range. To tackle this problem we performed a series of paired 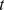-tests. Each test compares 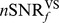 responses obtained for two different frequencies of stimulation for the group of subjects. The results shown in Fig. 5 were corrected for multiple comparisons by means of FDR (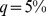). In this plot white pixel indicates a pair of stimulation frequencies (one from horizontal, and one from vertical axis) which do not yield significant differences in mean nSNR response, black pixel indicates that the difference is significant. One can notice, that there are contiguous, partially overlapping, frequency ranges which pairwise have equal mean responses. We selected four ranges of stimulation frequencies which are large and contiguous, marked by color squares. The next step was to find out which of these contiguous frequency range has the highest nSNR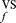 to fulfill our optimality criterion. One way ANOVA reveals that the mean nSNR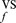 in these ranges are not equal (F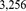 = 33.6, p = 2e–18). According to post-hoc test (Tukey HSD) we can state that at the significance level 5%(corrected) the mean nSNR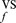 are different between each pair of the ranges (Fig. 6) except 8–14 Hz and 18–23 Hz. Thus the frequency bands can be ordered according to mean nSNR value in the following way: 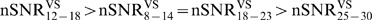


## Discussion

### Realistic design of the experiment

#### Paradigm

The experimental paradigms found in the SSVEP literature (e.g. [5], [6]) were limited to the long term stimulation with randomly selected frequencies, separated by short breaks. Such conditions are not realistic in a BCI system. The typical mode of operation of a BCI system consists of periods, when the subject focuses attention on the flickering light, and periods when he or she observes the BCI feedback or results of the selected action. This is true for both synchronous and asynchronous BCI systems. In this respect, proposed paradigm composed of stimulation epochs (VS) interleaved with the resting periods (NVS) seems more appropriate.

#### Number of flickering fields

In spite of claiming to investigate the properties of the SSVEP with the aim of improving SSVEP-based BCI, most of the reported studies were based upon unrealistic paradigm where the measures of the SSVEP response concerned the EEG activity recorded during stimulation with only one flickering light source [3], [5], [6], [26], [27]. The main objective of the presented study is determination of the SSVEP frequency response in the experimental condition involving the possible interference of neighboring, relatively large, flickering fields. In contrast to the previous studies, results presented here were obtained with a real BCI appliance [13]. In the presented paradigm each filed flickered with different, randomly selected frequency. Some of the attended frequencies have greater chance to occur together with the unattended frequencies having one of the harmonics equal to the attended one. For example one may consider attended stimulus 24 Hz which can be presented with the unattended stimuli 6, 8, 12 Hz which have a harmonic equal to 24 Hz in contrast to attended stimulus 23 Hz which has no unattended frequency with harmonics equal 23 Hz. A priori, it could be assumed that the frequencies which have many possibilities to be stimulated simultaneously with harmonically related neighbors would evoke different SSVEP response than those which have no such possibilities. But for the tested BCI appliance and methods of SSVEP evaluation, there is no visible interference from the not attended harmonically related frequencies. Careful inspection of Fig. 4 and 5 shows that frequencies which have higher chance to occur together with harmonically related frequencies don't exhibit visibly stronger responses.

### Measuring the SSVEP response

#### Derivation selection

The issue of an electrode montage optimal for SSVEP measurement was not unequivocally solved so far. In order to identify the electrode with the best SSVEP response, different techniques were used by different authors, e.g. averaging the signals from occipital leads (O1, Oz, O2) [5] or searching for the best bipolar combination of the occipital lead and some other electrode [4]. Following Molina [28] we propose to measure the SSVEP response in the channel of CSP filtered signals 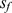 which corresponds to the greatest eigenvalue of 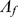. This channel is characterized by maximal variance for the time-series of interest and minimal variance for the time-series regarded as noise. The spatial patterns obtained for such selected channel are in most cases compatible with a presumed dipole in the visual cortex.

We showed that the selected spatial patten vary with stimulation frequencies (only four subject out of ten had the stable spatial pattern, independent of the stimulation frequencies). But even for the cases when the spatial patterns are independent of the stimulation frequencies, the spatial filters may vary with frequency due to different spatial distribution of background EEG activity in different frequency bands. These results suggest that the separate spatial filters should be estimated at least for the stimulation frequency ranges corresponding to different EEG rhythms.

#### Quantification of SSVEP

The response due to a flickering stimulation is mixed with the ongoing EEG activity. Thus the prerequisite to quantify the response is to separate it from the background activity. Measures of SSVEP proposed in [3], [5], [6] use time-locked averaging technique to suppress the background EEG activity. This method, although widely used in evoked potential research, is not very useful in a BCI setup, since it requires a sizable number of realizations to be effective.

The general signal processing techniques involving the frequency and spatial filtering to improve the signal-to-noise ratio can be used in the pre-processing stage, but at the next stage it is essential to construct a measure which is sensitive and specific to the activity resulting from the stimulation. There are two general approaches to quantification of the SSVEP. The first one is to use the amplitude of the spectral peak at the stimulation frequency. This can be obtained by autoregressive modeling [6] or Fourier analysis [4], [5]. The spectral amplitudes are then corrected for a typical EEG spectrum profile: e.g. by multiplying the results by the frequency [5]. The other one relies on constructing derived measures, e.g. the ratio of spectral power at the stimulation frequency to the average power in adjacent frequencies [4]. We showed that the range of frequencies which evoke most pronounced response depends to some extent on the applied response measure. For the population, average normalized spectral power measure 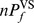 has maximum in the 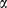 band while the 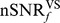 has the maximum in the low-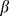 band. This discrepancy comes from the fact that the 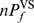 measure is affected by three factors: one is the activity induced by stimulation, the second is the attenuation of EEG amplitude with frequency, and the third one is the modulation of the background activity due to stimulation. The consequences of the second and third factors are reduced to great extent in case of the 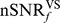 measure.

### Comparison of responses in different stimulating frequency

In this paper we investigated the responses to stimulation by light flickering with frequency in the 5–30 Hz range. The sensitivity of the brain to particular frequency as reflected by SSVEP frequency response curve was not uniform across frequencies and its exact shape depended on the method used to quantify the response, and on the subjects.

Our results are in agreement with other results reported in the literature, concerning SSVEP responses. Regan [3] has shown that the amplitudes of SSVEP exhibit resonant-like peaks in three frequency regions, with peak frequencies around 10 Hz, 20 Hz and 50 Hz. In our study only two first peaks are present as the range of frequencies investigated was limited to 5–30 Hz. It should be noted that these peaks are rather seen in individual subjects than in the group average. Pastor et al [5] has analyzed SSVEP responses to flicker stimulation in the 5–60 Hz range. They found that response reached a maximum at 15 Hz in occipital regions and at 25 Hz in frontal regions. The occipital maximum is in agreement with the one reported in our study, for 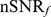 method. Analogous result was also presented for one subject in [4].

Herrmann [6] reported that the SSVEP responses exhibit resonance phenomena around 10, 20, 40 and 80 Hz. It has been shown in single subjects, by means of comparing responses to a given resonant frequency and two adjacent frequencies. Dominant responses at resonant frequencies were apparent in the power spectra and in their larger amplitude as compared to response amplitudes at both adjacent frequencies. In our study, the power spectra of the individual subjects don't point to the resonance properties of the brain, described above.

Of the two quantification methods studied in this paper the 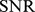 measure is more selective to the SSVEP phenomenon as explained above. Thus further statistical analysis were performed only for responses measured with 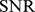. All the studied stimulation frequencies evoked response, yielding 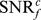 for VS epochs significantly higher than for NVS epochs. Using pair-wise tests we found contiguous, partially overlapping, frequency ranges with equal mean nSNR responses. The nSNR response, grouped in these ranges, have different group means. From the presented results it follows that the most effective frequencies evoking the largest SSVEP response belong to the frequency band of 12–18 Hz.
